# Cytokine signaling defects in primary atopic diseases—an updated review

**DOI:** 10.3389/falgy.2025.1617714

**Published:** 2025-07-01

**Authors:** Vaishali Thakur, Rakesh Kumar Pilania, Arunima Sharma, Saniya Sharma, Alfred Thomas Mario, Taru Goyal, Madhubala Sharma, Gayathri Coimbatore Vaitheeswaran, Pandiarajan Vignesh, Surjit Singh, Manpreet Dhaliwal, Amit Rawat

**Affiliations:** The Allergy Immunology Unit, Department of Pediatrics, Advanced Pediatrics Centre, Post Graduate Institute of Medical Education and Research, Chandigarh, India

**Keywords:** primary atopic disorders, monogenic allergic diseases, primary immunodeficiencies, inborn errors of immunity, allergy, atopy

## Abstract

Primary atopic disorders (PADs) are monogenic conditions associated with severe, early-onset atopic diseases. Clinically, they often overlap with polygenic allergic conditions, making specialized laboratory testing necessary to distinguish them from polygenic atopy. Multisystem involvement, such as growth failure, recurrent infections, and autoimmunity, points towards PADs warranting further investigations. PADs associated with immune dysregulation can be broadly categorized into four mechanistic groups: those affecting the regulation of cell cytoskeleton dynamics, T-cell receptor (TCR) signaling and repertoire diversity, and function of regulatory T cell (Treg), and cytokine signaling. In this review, we have examined the defects in cytokine signaling pathways associated with PADs. Key cytokine signaling pathways implicated in PADs include the STAT3, JAK1/STAT5b, and TGF-β pathways. Pathogenic variants in these pathways result in complex clinical phenotypes but share a common theme of Th2 polarization and severe atopic manifestations. Early and accurate differentiation between polygenic atopy and PADs is crucial, as it allows for timely, targeted immunological or genetic interventions that may significantly improve patient outcomes.

## 1 Introduction

Inborn errors of immunity (IEIs) are heritable disorders with a heterogenous presentation due to variants in genes impairing the activity of the immune system (1). Clinically, they may manifest as heightened susceptibility to infections, autoinflammation, autoimmunity, atopy, and malignancies (2). A subset of these IEIs features a distinct atopic phenotype marked by chronic Th2 skewing, aberrant mast cell degranulation, eosinophilic inflammation, and elevated IgE levels (3). In 2018, Lyons and Milner introduced the term primary atopic disorders (PADs) to categorize monogenic conditions associated with early-onset atopic symptoms driven by immune dysregulation (4). However, it is now recognized that PADs are not a strict subcategory of IEIs. Only a subset of PADs are immune in origin and may be classified as IEI. Many PADs result from non-immune mechanisms involving structural or barrier defects. These PADs primarily disrupt epithelial integrity, leading to heightened allergen penetration and subsequent atopic responses. Prototypic disorders of these PADs are attributed to variants in genes encoding epidermal barrier proteins like *FLG* (filaggrin), protease inhibitors such as *SPINK5*, and intercellular adhesion molecules *CDSN*, *DSG1*, and *DSP* (5–7).

**Figure 1:**
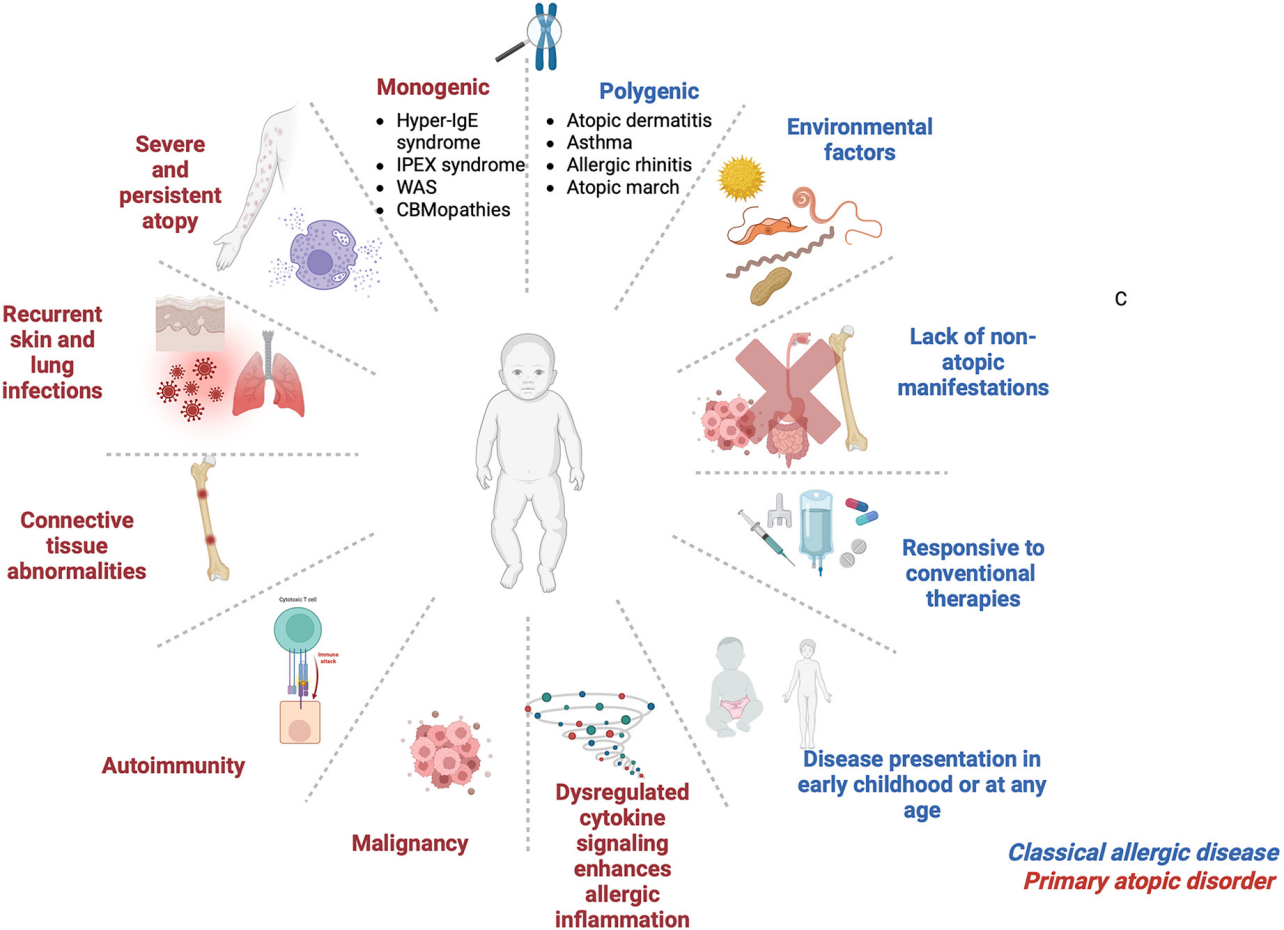
Clinical characteristics for classical allergic disease and primary atopic disorder. IPEX, Immune dysregulation, polyendocrinopathy, enteropathy, X-linked; WAS, Wiskott–Aldrich syndrome; CBMopathies, CBM complex—*CARD11*, *BCL10* and *MALT1*. Figure was created using BioRender.

IEIs with atopic manifestations exhibit overlapping clinical and immunological phenotypes and have been grouped into key syndromic categories. These are classified based on clinical presentation and immunological profiling as Hyper-IgE syndromes, immune dysregulation poly-endocrinopathy enteropathy X-linked (IPEX) and IPEX-like conditions, Omenn syndrome, Wiskott–Aldrich syndrome, CBM-opathies, and other atopy predominant IEIs (8). Although clinical features of PADs often overlap with polygenic allergic conditions, they are distinguished by early onset and severe manifestations with complex comorbidities such as growth failure, recurrent infections, and autoimmunity, to name a few (9) (Figure 1).

Atopy is a genetic tendency to mount exaggerated IgE-mediated immune responses to environmental allergens. These patients often present with a constellation of clinical symptoms, such as atopic dermatitis, food allergy, allergic rhinitis, and asthma - collectively referred to as the ‘atopic march’ (10–13). In PADs, these manifestations are often accompanied by immune dysregulation due to Th2 polarization and overproduction of IL-4, IL-5, and IL-13. These Th2 effector cytokines drive downstream signaling cascades that recruit eosinophils, mast cells, and other effector cells (11). While allergic phenotypes are common in the general population, those seen in IEIs are frequently more severe and are rooted in well-defined genetic defects (9).

**Figure 2:**
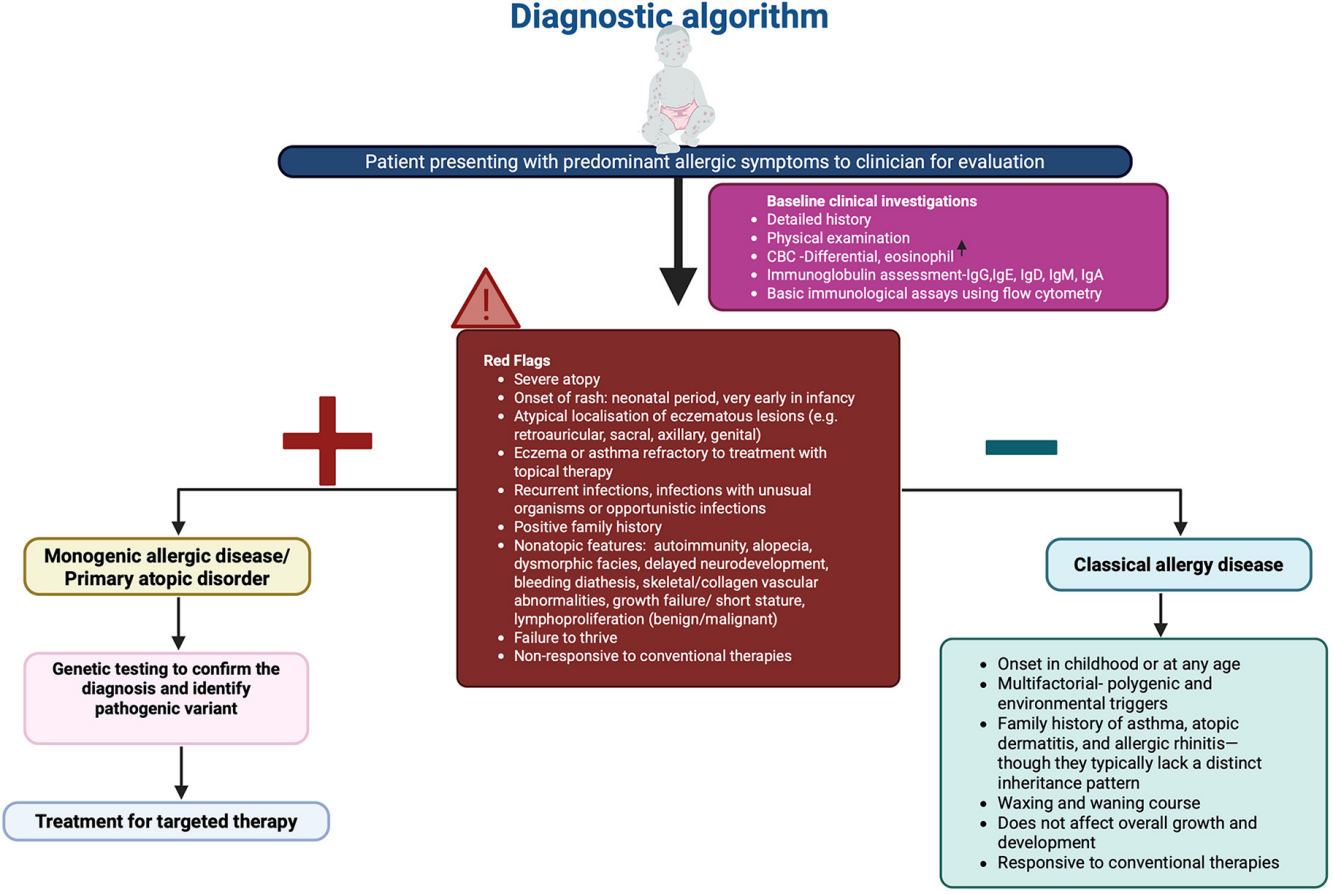
Diagnostic algorithm in patients with suspected primary atopic disorder. Figure was created using BioRender.

Diagnosing PADs begins with a thorough clinical evaluation, including physical examination, family history, and immunological investigations (14) (Figure 2). Family medical history is important as PADs typically exhibit distinct inheritance patterns, although de-novo variants may arise spontaneously as well. Hallmark laboratory findings include eosinophilia, elevated serum IgE, abnormal immunoglobulin profiles, and T-cell subsets, which are typically assessed via flow cytometry (14). Genetic testing plays a pivotal role in confirming the diagnosis by identifying causal variants (15).

Key immune pathways involved include those regulating the cellular cytoskeleton along with immune synapse formation, T-cell receptor (TCR) signaling and repertoire diversity, T regulatory cell (Treg) function, and innate immune cell effector mechanisms (16). Among the molecular mechanisms implicated in PADs, signaling pathways of cytokines have a cardinal role. Disruption in cytokine signaling can severely impair host immune response and tolerance, predisposing individuals to infections, inflammation, and allergic diseases (17). Specifically, alterations in STAT3, JAK1/STAT5, and TGF-β pathways through molecular consequences, such as dominant-negative (DN), loss or gain-of-function (LOF, GOF) mutations, are closely associated with Th2 polarization and atopic manifestations (14).

In this review, we focus on PADs arising from genetic defects that disrupt cytokine signaling networks. We examine mutations affecting transcription factors such as *STAT1*, *STAT3*, *STAT5B, STAT6, ZNF341*, cell surface receptors including *IL2RA, IL4RA, IL6R, IL6ST*, *TGFBR1/2*, and intracellular signaling mediators such as *JAK1* and *ERBB2IP* (18)*.* Understanding these pathways not only enhances our insight into the pathogenesis of PADs but also provides a foundation for developing precision medicine. The key primary atopic disorders resulting from cytokine signaling defects have been summarised in Table 1.

**Table 1:** Primary atopic disorders resulting from cytokine signaling defects.

S.No.	Gene	GOF/LOF molecular consequences	OMIM genotype no	OMIM phenotype	Inheritance pattern	Associated clinical phenotype	Targeted therapy
1	*STAT 1*	GOF	*600555	614162	AD	CMC, autoimmunity, type I interferonopathies, IPEX-like syndrome	Ruxolitinib, (75, 76) HSCT (77)
2	*STAT3*	DN	*102582	147060	AD	HIES, eczema, recurrent skin and lung infections, eosinophilia, candidiasis, skeletal and connective tissue abnormalities retained primary teeth, characteristic facies, cerebral aneurysms	Tocilizumab and JAK inhibitors, (78) chaperone modulators like HSF1A and GGA, offering a promising future treatment, HSCT (78, 79)
3	*STAT5B*	GOF	*604260	AR-245590 AD-618985	Somatic	Hypereosinophilic syndrome, growth failure, immune dysregulation, and lymphoproliferation.	JAK inhibitors (Ruxolitinib (80)
4	*STAT5B*	LOF	*604260		AD, AR	Eczema, IPEX-like autoimmune manifestations Autosomal dominant form causes dermatitis but without severe immunodeficiency	Cyclosporine therapy (81)
5	*STAT6*	GOF	*601512	620532	AD	Severe and treatment-resistant dermatitis, marked eosinophilic gastrointestinal disease	Ruxolitinib Tofacitinib Dupilumab (41)
6	*ZNF341*	LOF	*618269	618282	AR	Phenocopy of STAT3 DN	Dupilumab (82)
7	*IL2RA*	LOF	*147730	606367	AR	IPEX-like syndrome (e.g., enteropathy, endocrinopathies, and failure to thrive)	Rapamycin (83)
8	*IL4RA*	GOF	*147781		AD	Early-onset atopic dermatitis, hyper IgE levels, food allergies, asthma and autoimmunity	Dupilumab (51)
9	*IL6ST* (Partial) *IL6ST* (Complete)	LOF	*600694 *600694	619752 619751	AD AR	Phenotypic overlap with AD-HIES Stuve-Wiedemann-like syndrome	Supportive treatment (54)
10	*IL6R*	LOF	*147880	618944	AR	Partially overlapping with AD-HIES: No connective tissue abnormalities	Immunoglobulin replacement therapy (84), HSCT (85)
11	*TGFBR1 TGFBR 2*	LOF	*190181 *190182	609192 610168	AD	Marfan-like syndrome; phenotypic overlap with STAT3 pathway disorders	HSCT surgical interventions (86)
12	*JAK1*	GOF	*147795	618999	AD	Hypereosinophilic syndrome	JAK inhibitors like ruxolitinib and tofacitinib (65, 66)
13	*ERBIN*	LOF	*606944		AD	Significant eosinophilic esophagitis, cutaneous mastocytosis, connective tissue abnormalities	Dupilumab (87)

GOF, gain of function; LOF, loss of function; AD, autosomal dominant; AR, autosomal recessive; IPEX, immune dysregulation, polyendocrinopathy, enteropathy, X-linked; HSCT, hematopoietic stem cell transplant; GGA, geranylgeranylacetone; JAK, janus kinases; EGID, eosinophilic gastrointestinal disease.

## 2 Role of transcription factors in altered cytokine signaling

### 2.1 STAT1 GOF

Autosomal dominant- GOF variants in *STAT1* are among the commonest monogenic defects linked to chronic mucocutaneous candidiasis (CMC), with over 400 reported cases (19, 20). These mutations prevent dephosphorylation of STAT1, leading to its constitutive nuclear localization and enhanced type I/II interferon signaling (21). Consequently, Th17 differentiation is impaired, which directly impacts antifungal defenses, and enhanced interferon signaling drives autoimmunity (e.g., hypothyroidism, cytopenias) that resemble IPEX-like phenotypes (22). A novel N-terminal mutation (c.194A>C; p.D65A) has even been tied to eosinophilic esophagitis, underscoring the link between STAT1 hyperactivity and atopic inflammation (21, 23).

### 2.2 STAT3 DN

First described clinically as Job's syndrome in 1966, autosomal -dominant DN mutations in *STAT3* are the molecular basis of Hyper -IgE syndrome (HIES) (24–26). Patients exhibit severe eczema, elevated IgE (>1,000 IU/ml), eosinophilia, and recurrent staphylococcal skin and pulmonary infections (27, 28). Dampened IL-6 and IL-10 signaling through STAT3 reduces Th17 cell numbers and IL-17 production, heightening susceptibility to *Staphylococcus* and *Candida* infections (27, 29). Diagnostic criteria combine IgE quantification, Th17 enumeration, a clinical scoring system (>30 points), and genetic confirmation of a *STAT3* DN variant (30).

### 2.3 STAT5B GOF

Somatic GOF variants in the SH2 or transactivation domains of *STAT5B* enhance STAT5 signaling, driving clonal T-cell expansion with a Th2 bias (31). Thus, STAT5B remains constitutively active instead of responding appropriately to growth hormone signals that regulate IGF-1–dependent growth but also skew T-cell differentiation towards a Th2 phenotype. Clinically, affected individuals present with treatment-refractory atopic dermatitis, persistent urticaria, elevated numbers of eosinophilia, alopecia, and angioedema (32).

### 2.4 STAT5B LOF

*STAT5B* loss-of-function leads to atopic dermatitis with dwarfism, hyper IgE, autoimmunity and lymphocytic interstitial pneumonitis (33, 34). *STAT5B* acts as a key mediator in growth hormone signaling, and its deficiency results in growth hormone insensitivity and growth failure (35). Additionally, STAT5B LOF impairs IL-2-dependent signaling, leading to recurrent viral infections associated with reduced function and even decreased numbers of T regs (34). Atopic symptoms such as eczema are prevalent and affected individuals may develop conditions resembling IPEX like syndrome (33). In addition to LOF variants, the STAT5B deficiency can also result from the autosomal dominant form of *STAT5B,* causing stunted growth and eczema, but it does not lead to severe immunodeficiency (36).

### 2.5 STAT6 GOF

STAT6 is the major transcription factor activated by IL-4 and IL-13. Upon activation, STAT6 dimerizes and translocates to the nucleus. STAT6 promotes differentiation of naive CD4^+^ T cells to Th2 cells along with class-switch from IgM to IgE on B-cells (37). This process is initiated on binding of IL-4 and IL-13 to the IL-4 receptor complex, triggering the phosphorylation of tyrosine residues on the IL-4 receptor alpha (IL-4Rα) subunit (38). This is mediated via Janus kinases (JAK) (39). Src homology 2 (SH2) domains recruit STAT6, which bind to the phosphorylated tyrosine residues on IL-4Rα (39). In *STAT6* GOF, hyperphosphorylation of STAT6 intensifies IL-4 and IL-13 signaling. A *STAT6* heterozygous misense variant (c.1129G>A; p.Glu377Lys) linked to atopy was initially identified by Suratannon et al. (40). Another missense variant in exon 22 (c.1114G>A; p.E372K) was identified in a patient with early-onset eczema, food allergies, eosinophilia, and eosinophilic esophagitis (41). Functional studies demonstrated heightened IL-4/IL-13 responsiveness, reversed by JAK inhibition (ruxolitinib) or IL-4Rα blockade, which normalized IgE levels and tissue eosinophilia (41). To date, STAT6-GOF mutations have been reported in 21 persons (42).

### 2.6 ZNF341 LOF

ZNF341 is a zinc-finger transcription factor that upregulates both STAT1 and STAT3 expression (43). Autosomal-recessive LOF mutations in *ZNF341* phenocopy STAT3-HIES, causing elevated IgE, eosinophilia, eczema, and recurrent bacterial and fungal infections (43, 44). Till now, 20 patients with autosomal recessive LOF ZNF341 have been reported (44). Unlike STAT3 DN, connective tissue defects tend to be milder, but the underlying mechanism, diminished STAT3 transcription remains the same.

## 3 Cell surface receptor defects in altered cytokine signaling

### 3.1 IL-2Rα (CD25) LOF

The α-chain of the high-affinity IL-2 receptor is encoded by *IL2RA* and is essential for regulatory T-cell development and peripheral tolerance (45). Biallelic loss-of-function mutations in *IL2RA* produce an IPEX-like syndrome characterized by severe atopic dermatitis, eosinophilia, elevated IgE, autoimmunity, and chronic infections (22, 46). Defective IL-2 signaling impairs Treg homeostasis, which results in unchecked Th2 and Th17 responses that drive both allergic and autoimmune pathology (22).

### 3.2 *IL-4RA* GOF

Gain-of-function (GOF) variants in *IL4RA*, particularly the R576 allele, are strongly associated with increased susceptibility to atopic diseases (47). The Q576R variant in IL-4RA disrupts the formation of the STAT3–ERBIN–SMAD2/3 complex (48). Impaired STAT3 and ERBIN function intensifies Th2 polarization by reducing TGF-β signaling and increasing IL-4RA expression on lymphocytes (49). These changes culminate in a clinical phenotype characterised by early-onset atopic dermatitis, elevated serum IgE, asthma, food allergies, and, in some cases, autoimmune features (50). Importantly, dupilumab, an IL-4Rα antagonist, has demonstrated clinical efficacy in treating patients with variants in IL-4RA (51).

### 3.3 IL6ST LOF

*IL6ST* encodes GP130, the shared signal-transducing subunit for all IL-6 family cytokines. The main cytokines of IL-6 family include IL-6, IL-11, IL-27, IL-35, IL-39, and oncostatin M (52, 53). Recessive LOF variants abolish JAK/STAT3 activation, manifesting as an autosomal-recessive Hyper-IgE syndrome with eczema, high IgE, eosinophilia, and recurrent bacterial infections (54). Recently, dominant-negative *IL6ST* mutations (c.2261C>A, p.Ser754Ter) have been linked to autosomal-dominant HIES phenotypes (54, 55), and secondary glycosylation defects (e.g., in PGM3 deficiency) can similarly impair GP130 surface expression and STAT3 phosphorylation (56).

### 3.4 IL-6R LOF

One of the key functions of IL-6 signaling is to differentiate activated Th cells into IL-17 and IL-22 secreting Th17 and Th22 cells (53).). On the other hand, IL-6 suppresses the differentiation of CD4+ T regulatory cells, which regulate inflammation. Individuals deficient in IL-6R develop atopic dermatitis, eosinophilia, recurring pulmonary infections, skin abscesses due to *Staphylococcus* sp., high IgE levels, but no skeletal abnormalities (57, 58).

### 3.5 TGF-β receptor (TGFBR1/2) deficiency in Loeys–Dietz syndrome

Heterozygous mutations in *TGFBR1* or *TGFBR2* cause Loeys–Dietz syndrome (59). This is an autosomal-dominant connective-tissue disorder marked by arterial aneurysms, craniofacial abnormalities, and severe atopic features in the form of asthma, food allergy, and eosinophilic gastrointestinal disease (60). The TGFBR1/2 complex recognises TGF-β, and variants in the receptor may lead to dysregulated TGF-β signaling, which may enhance SMAD2/3 phosphorylation (61). This results in conversion of a tolerogenic pathway to a pro-allergic one by producing dysfunctional Tregs and by enhancing transcription of IL-9 and other pro-allergic mediators (62).

## 4 Key defects in intracellular signaling components leading to altered cytokine signaling defects

### 4.1 JAK1 GOF

Germline *JAK1* GOF mutations lead to constitutive activation of the Janus kinase 1 protein, leading to immune dysregulation due to unchecked STAT phosphorylation (63). Mechanistically, JAK1 hyperactivity skews CD4^+^ T-cell differentiation toward a Th2 phenotype while suppressing Th1 responses (63). This results in amplification of allergic inflammation (63). These variants lead to novel monogenic immune dysregulation syndrome, termed JAACD**—**JAK1-associated Autoimmunity, Atopy, Colitis, and Dermatitis. Affected individuals exhibit a syndromic phenotype characterized by early-onset atopic disease, autoimmune features, severe dermatitis, and inflammatory bowel manifestations such as colitis (64). Del Bel et al. described the first germline GOF mutation in humans in JAK1, resulting in an alanine to aspartate substitution at position 634 (65). Three more variants were identified in *JAK1*, namely, S703I, H596D, and C787F, from patients with a similar clinical phenotype (66–68). Recently, Horesh et al. described 59 patients with JAACD spectrum harbouring four JAK1-GOF variants (p.E139K, p.R506C, p.S700N, and p.V985I). These patients share a common phenotype of severe atopy with autoimmunity and immune dysregulation (64).

### 4.2 ERBB2IP (ERBIN) LOF

*ERBB2IP* encodes ERBIN, a scaffold protein that links activated STAT3 to SMAD2/3 complexes, sequestering them in the cytoplasm and thereby restricting TGF-β signaling (49, 69). Impaired STAT3 signaling can decrease ERBIN levels resulting in disrupted regulation of TGF-β and consequent increase in Tregs. Autosomal-recessive LOF variants in *ERBB2IP* disrupt this regulator*y* axis, leading to excessive SMAD2/3 nuclear translocation, enhanced Treg proliferation, and paradoxical Th2 polarization (49). Clinically, ERBIN-deficient patients exhibit anomalies reminiscent of STAT3-HIES, despite a distinct molecular etiology. Patients may present with severe atopic dermatitis, eosinophilic gastrointestinal diseases, elevated IgE, and connective-tissue disorders. Emerging biologic therapy, such as IL-4Rα blockade with dupilumab has shown promise in reducing Th2-driven inflammation in this disorder (70).

## 5 Future perspectives

Next-generation sequencing (NGS) is revolutionizing the diagnosis of primary atopic disorders (PADs) by enabling rapid identification of disease-causing variants. This technology is especially valuable in patients with complex or treatment-refractory presentations, where it can resolve long-standing diagnostic challenges. However, as the use of NGS expands, a growing number of variants of uncertain significance (VUS) or cases lacking identifiable pathogenic variants are being reported. These findings often complicate clinical decision-making and may necessitate extensive additional testing.

Large-scale analyses of genetic testing across hereditary diseases have underscored the growing burden of variants of uncertain significance (VUS). In a study involving over 1.6 million individuals, 41% had at least one VUS, and nearly one-third received only VUS results (71). Despite efforts, only 7% of unique VUSs were reclassified as pathogenic or likely pathogenic, often taking over two years (71). These findings underscore the broader systemic challenge posed by VUSs across rare disease diagnostics and highlight the need for structured interpretive frameworks that could also benefit the PAD diagnostic landscape.

Looking forward, decision-making around functional validation of variants must be guided by integrated criteria, including in silico prediction tools, family segregation analysis, phenotypic concordance, and population frequency data (72). While functional assays remain the gold standard for confirming pathogenicity, they are resource-intensive and often inaccessible in routine clinical settings.

Future diagnostic frameworks must prioritise variants with the highest clinical relevance. Innovative tools such as multiplexed assays of variant effect (MAVEs) offer a promising, high-throughput approach to functional validation (73). Concurrently, emerging efforts to harmonise the interpretation of single-nucleotide variants (SNVs) and copy-number variants (CNVs) are streamlining variant classification in rare diseases (74).

To fully realise the promise of precision medicine in PADs, future efforts should focus on building standardised, scalable, and integrative diagnostic pipelines. Such systems will be essential for accelerating diagnosis, guiding targeted therapies, and ultimately improving clinical outcomes for patients.

## 6 Conclusion

PADs represent a critical intersection between monogenic immune dysregulation and severe allergic inflammation. Cytokine signaling defects involving JAK-STAT and TGF-β pathways can result in profound allergic phenotypes often misdiagnosed as common atopy. Timely diagnosis of the pathogenetic defect using advanced next-generation sequencing is essential to deliver targeted, immune-based therapies. As our understanding of PADs continues to expand, personalized approach will eventually be the standard of care for affected individuals.
